# Why are only some children with autism spectrum disorder misclassified by the social communication questionnaire? An empirical investigation of individual differences in sensitivity and specificity in a clinic-referred sample

**DOI:** 10.1186/s11689-023-09497-7

**Published:** 2023-08-22

**Authors:** Chimei M. Lee, Melody R. Altschuler, Amy N. Esler, Catherine A. Burrows, Rebekah L. Hudock

**Affiliations:** 1 Division of Clinical Behavioral Neuroscience, Department of Pediatrics, University of Minnesota, 2025 E River Pkwy, Minneapolis, MN 55414 USA; 2 Department of Psychology, Berea College, 101 Chestnut St, Berea, KY USA

**Keywords:** Autism spectrum disorder, Social Communication Questionnaire, Sensitivity, Specificity, Individual differences, Screening, Internalizing, Externalizing, Adaptive behavior, Diagnosis, Early identification

## Abstract

**Background:**

The Social Communication Questionnaire (SCQ) is a checklist for autism spectrum disorder (ASD) commonly used in research and clinical practice. While the original validation study suggested that the SCQ was an accurate ASD screener with satisfactory sensitivity and specificity, subsequent studies have yielded mixed results, with some revealing low sensitivity, low specificity, and low utility in some settings.

**Method:**

The present study examined the psychometric properties of the SCQ as well as the individual difference characteristics of 187 individuals with and without autism spectrum disorder (ASD) who were misclassified or accurately classified by the SCQ in a clinic-referred sample.

**Results:**

The SCQ showed suboptimal sensitivity and specificity, regardless of age and sex. Compared to true positives, individuals classified as false positives displayed greater externalizing and internalizing problems, whereas individuals classified as false negatives displayed better social communication and adaptive skills.

**Conclusions:**

The findings suggest that non-autistic developmental and behavioral individual difference characteristics may explain high rates of misclassification using the SCQ. Clinicians and researchers could consider using the SCQ in combination with other tools for young children with internalizing and externalizing symptoms and other more complex clinical presentations.

Heterogeneity in symptom presentation makes accurate screening and assessing for autism spectrum disorder (ASD) particularly challenging. While a variety of measures have been developed to identify children at risk for ASD, a commonly used tool in research and clinical practice is the Social Communication Questionnaire (SCQ, formerly named Autism Screening Questionnaire) [1]. The SCQ was developed to be a reliable and valid screener for ASD [2]. It has been widely used in the clinical setting for triage and referral decision-making, as well as in research settings to serve as a screening tool for inclusion or exclusion criteria. Studies examining the SCQ have yielded mixed results, with some suggesting low sensitivity, low specificity, and low utility within certain clinical settings due to its unsatisfactory psychometric properties [3–9]. Given the recent literature suggesting that the SCQ may not be uniformly useful as a screening tool for ASD, research is needed to better understand factors that might be affecting SCQ’s sensitivity and specificity.

In a recent meta-analysis that systematically assessed the accuracy of the SCQ over the last 15 years, the authors concluded that variability in sampling methods impacted the accuracy of the SCQ for ASD screening [10]. Indeed, the SCQ had significantly higher sensitivity and specificity in samples that were population reference samples compared to clinical samples, community samples, and convenience samples, and the effect size was the biggest when comparing population samples to clinical samples [11]. Moreover, a recent study of a clinic-referred sample of verbally fluent children and adolescents found that the SCQ performed no better than chance in correctly classifying children with ASD and those with non-spectrum diagnoses [3]. The poor performance of the SCQ in accurately classifying ASD among clinical samples further suggested that individual characteristics may influence variability in the SCQ’s effectiveness as a screening tool [12, 13].

An emerging literature suggests that individual variables that may explain variability in SCQ effectiveness include the demographic factors of age and sex. Several studies reveal lower accuracy for the suggested SCQ cut-offs at younger ages. Maintaining a cut-off of 15, the SCQ has demonstrated satisfactory results in school-age populations [5, 14]; however, the sensitivity and specificity hover between 0.50 to 0.75 for younger populations [4, 5, 7, 10]. For children under 8 years old, Corsello et al. [5] also reported low sensitivity and specificity for the published cut-off of 15 and suggested lowering the SCQ cut-off score to ≥ 11 (for age under 5) or ≥ 12 (for age 5–7) to achieve better sensitivity. Subsequent studies confirmed a cut-off score of 11 maximizes sensitivity and specificity (a better balance between these two critical indices) in young children [13, 15–17]. Another study found that the recommended cut-off of 15 had accurate sensitivity and specificity for adolescents and adults (ages 13–21), but a cut-off of 11 worked best for children ages 4 to 12 years [11].

Sex differences have also been found on assessment measures for ASD [18, 19], although only a few studies have examined sex differences in SCQ accuracy. Evans and colleagues found that the SCQ performed adequately in boys (sensitivity 0.77; specificity 0.66) and well in girls (sensitivity 1; specificity 0.71) among 272 school-age children [20]. However, in a multisite case-control study of young children with either ASD or non-spectrum developmental disabilities, males scored significantly higher than females on the SCQ in both groups [15]. Several studies did not compare sensitivity and specificity directly but found similar SCQ mean total scores for males and females [2, 3, 5, 13]. Overall, extant research examining the impact of age and sex on SCQ accuracy has produced conflicting results, and work is needed to understand whether these demographic factors impact the effectiveness of SCQ as a screening tool.

In addition to demographic characteristics, individual differences in clinical presentations may also influence variability in the SCQ results [12, 13]. Previous studies have continued to find poor sensitivity–specificity balance in distinguishing between ASD and other developmental disorders among children [21, 22]. Suren and colleagues [23] found that sensitivity of the SCQ was higher for children with ASD who had not developed phrase speech and were significantly delayed in their cognitive development. Similarly, other studies have revealed satisfactory sensitivity (ranging from 0.88 to 1.00) but poor specificity (ranging from 0.17 to 0.43) in distinguishing between individuals with intellectual disabilities with and without ASD [24, 25]. Multiple studies have found a negative correlation between SCQ total scores and adaptive skills and intelligence quotient (IQ) [7, 9, 26, 27]. Findings suggest that parent report on the SCQ is related to individual differences in children’s cognitive ability, language ability, and adaptive functioning.

Internalizing and externalizing psychopathology are also candidate factors for explaining variation in SCQ effectiveness. Studies examining the clinical utility of the SCQ in samples of children with a variety of neurodevelopmental and mental health diagnoses generally have found lower sensitivity and specificity than in the original validation study [3, 7, 26, 27]. For example, using a heterogenous sample, Hollocks et al. [3] found generally adequate sensitivity when using the SCQ cut-offs ranging from 13 to 20, but specificity below 0.40 for all cut-offs (the recommended cut-off of 15 had a sensitivity of 0.84 and specificity of 0.13). Ung et al. [26] also reported suboptimal sensitivity (0.70) and specificity (0.67) at the recommended cut-off of 15, and a cut-off of 11 improved sensitivity to 0.82, at a cost to specificity (0.37). Moreover, studies comparing children with ASD and attention-deficit hyperactivity disorder (ADHD) have found adequate sensitivity and specificity of the SCQ [28, 29], while one study found the optimal cut-off of the SCQ was lower than the recommended cut-off of 15 for differentiating ASD from ADHD [28]. These findings suggested that the SCQ scores are highly influenced by non-autism developmental and behavioral factors, which further raise questions as to whether sensitivity and specificity of the SCQ are adequate for screening purposes.

Taken together, individual differences in clinical presentation, such as cognitive ability, language ability, comorbidities, and the presence of challenging behaviors seem to be impacting the effectiveness of the SCQ. Other factors including sample referral source, sample heterogeneity, and parental understanding of ASD may also impact the performance of the SCQ. Studies to date have identified individual difference factors described above as broad categories of features that can influence the accuracy of SCQ, but more research is needed on the specific behavioral and developmental features that impact accuracy, especially those that cross diagnostic categories.

## Present study

The current study aims to overcome the limitations of prior research by focusing on a heterogeneous group of children from a clinic-referred sample and examining whether individual difference characteristics differentiate those who are misclassified by the SCQ. The first aim of the present study adds to the literature on SCQ accuracy by estimating its sensitivity and specificity in a large clinical-referred sample. Based on prior literature, we hypothesize that the SCQ will show lower sensitivity and specificity than that presented in the original validation study. The second aim is to examine the individual characteristics of children misclassified by the SCQ. We hypothesize that the presence of more comorbid conditions, lower cognitive ability, greater autism severity, greater internalizing and externalizing symptoms, and lower adaptive functioning may explain why the sensitivity and specificity of the SCQ are poor in clinic-referred samples. The results of the study provide valuable information when considering using the SCQ in a clinical or research setting, especially for a population who are seeking their first-time ASD diagnosis or assessment.

## Methods

### Participants

Participants included individuals who received diagnostic neurodevelopmental evaluations at an autism specialty clinic, referred by neontologists, pediatricians, geneticists, pediatric neuropsychologists, psychiatrists, as well as other professionals who work closely with the families (i.e., speech pathologists, occupational therapists). Clinical diagnoses were made by licensed psychologists with extensive experience in the assessment and treatment of ASD and common co-occurring conditions (5–15 years of clinical experience). Licensed psychologists integrated multiple sources of information, including medical history, educational records, caregiver reports, neuropsychological assessments, and comprehensive interviews regarding autistic characteristics to make diagnostic decisions based on DSM-5 criteria. The SCQ was collected as part of the diagnostic evaluation but was not the sole factor considered when making a diagnosis. The methods were approved by the Institutional Review Board at the University of Minnesota.

From 2016 to 2019, data from 793 consecutive evaluations were entered into a de-identified clinical database. To examine the current study hypotheses, only initial evaluation data (*N* = 519) were included in the analysis. Data of individuals who were younger than 4 years old at their initial evaluations (*N* = 142) were excluded from analyses. Individuals who did not complete or only partially completed the SCQ (*N* = 162) were also excluded. Finally, only participants who had completed all the measures used in the present study (described below) were included. Ultimately, initial diagnostic evaluations from 187 children and adolescents were included in the present study.

Among all individuals, 133 (94 males and 39 females) were clinically diagnosed with ASD, and 54 (34 males and 20 females) were identified as non-spectrum with other neurodevelopmental challenges. Within the non-spectrum sample, 32 received a primary diagnosis of ADHD (59.3%), 10 were diagnosed with a language disorder (18.5%), 5 had behavioral disorders (9.3%; e.g., oppositional defiant disorder, conduct disorder, disruptive behavioral disorder), 27 had anxiety disorders (50.0%), 7 had mood disorders (13.0%), and 8 had other genetic and/or physical disabilities (14.8%; e.g., fragile X syndrome, Williams syndrome, or mild cerebral palsy).

### Measures

Demographic characteristics were collected through a clinical intake questionnaire and clinical interviews with the primary caregivers. All participants were given the Autism Diagnostic Observation Schedule, Second Edition (ADOS-2), and caregivers were administered the Autism Diagnostic Interview-Revised (ADI-R). Cognitive and language skills were assessed using clinically appropriate measures depending on the child's age and function level. Scores on individual sub-tests are standardized against age-specific norms and then grouped to produce separate measures of verbal and non-verbal IQ, with the former encompassing those tests most related to verbal skills and the latter being more independent of verbal skills. Full Scale IQ (FSIQ) is the composite of these verbal and non-verbal skills. All individuals also completed the following measures.

#### Social Communication Questionnaire, Lifetime Version (SCQ)

The SCQ [1] is a 40-item questionnaire that measures the symptomatology associated with ASD (e.g., certain communitive behaviors, language uses, and stereotyped behaviors) focusing on the behaviors that are rare in non-affected individuals, based on an established diagnostic interview, the Autism Diagnostic Interview-Revised (ADI-R) [30]. The Lifetime SCQ version asks respondents to focus on characteristics of the individual at age 4 to 5 years for developmentally influenced behaviors, or at any point in their lifetime for behaviors that are atypical at any age (e.g., repetitive motor movements), while the Current SCQ version focuses on characteristics present within the previous 3 months [1]. Total scores range from 0 to 39, with higher scores reflecting the presence of more symptoms.

#### Behavioral Assessment System for Children, Parent Rating Scale (BASC-PRS)

The BASC-PRS [31] is a parent-report questionnaire using a multi-dimensional approach to evaluate behaviors and adaptive skills for children ages 2 years, 6 months to 21 years. This sample completed the BASC-PRS, Third Edition, which generates four composite scales (Externalizing Problems, Internalizing Problems, Behavioral Symptoms Index, and Adaptive Skills) and 14 Primary Scales (Hyperactivity, Aggression, Conduct Problems, Anxiety, Depression, Somatization, Atypicality, Withdrawal, Attention Problems, Adaptability, Social Skills, Leadership, Activities of Daily Living, and Functional Communication). For behavioral scales, higher T scores indicate greater difficulties, whereas for adaptive scales, lower T scores represent greater challenges [31].

#### Vineland Adaptive Behavior Scale (VABS)

The VABS [32, 33] is a standardized clinical assessment tool that utilizes a semi-structured interview to measure adaptive behaviors and skills for individuals with developmental challenges. For this sample, the third edition of the VABS, Comprehensive Interview Form, was used to assess participants’ adaptive skills. The VABS consists of three subscales, including Communication (receptive, expressive, written), Socialization (interpersonal relationships, play and leisure, coping skills), and Daily Living skills (person, domestic, community), yielding an overall composite score of adaptive skills (Adaptive Behavior Composite). The VABS also provides an indirect measure of gross and fine motor skills, yielding a Motor skills domain [32, 33].

#### Autism Diagnostic Observation Schedule, Second Edition (ADOS-2)

The ADOS-2 [34] is a standardized, semi-structured observational assessment used to assess language and communication, reciprocal social interaction, imagination/creativity, as well as stereotyped behaviors and restricted interests to inform diagnosis of ASD. The ADOS-2 is organized into five modules based on the individual’s expressive language level (and, in some cases, chronological age), ranging from preverbal to verbally fluent. The diagnostic algorithm provides separate total scores for the Social Affect (SA) and Restricted and Repetitive Behavior (RRB) domains, as well as a cut-off for the sum of the two domains to provide instrument classifications of autism, autism spectrum, or non-spectrum. The Calibrated Severity Score (CSS) is a standardized version of ADOS-2 raw total scores aimed to minimize the impact of factors such as age, language, and cognitive ability. The ADOS-2 CSS has been suggested as a measure of symptom severity independent of these developmental factors [35].

### Analytic approach

Data collected through the comprehensive evaluations were entered, coded, and checked for errors and logic using a standardized procedure. Subsequently, the entered data were transferred to SAS 9.4 (SAS Institute Inc., Cary, NC, USA) to perform range checking and internal consistency checking. Based on the clinical diagnosis of ASD and suggested cut-off score of the SCQ (summed score of 15 or above) [1], individuals were separated into four groups to test our hypotheses: true positives (TP; have a clinical diagnosis of ASD and the SCQ score is above 15;* N* = 82), false negatives (FN; have a clinical diagnosis of ASD but the SCQ score is below 15;* N* = 51), false positives (FP; does not have a clinical diagnosis of ASD but the SCQ score is above 15;* N* = 22), and true negatives (TN; does not have a clinical diagnosis of ASD and the SCQ score is below 15; *N* = 32). Demographic homogeneity of the groups was assessed using chi-square tests and post-hoc analyses (Fisher’s exact test) for discrete variables. Continuous variables, such as sum or mean score of cognitive measures, symptomatologic items, as well as emotional, behavioral, and adaptive measures, were compared between groups by using independent *t* tests and post-hoc analyses (Bonferroni correction). To determine the performance of the SCQ as a screener for ASD in this clinical-referred sample, the sensitivity (i.e., number of true positive/[true positive + false negative]) and specificity (i.e., number of true negative/[true negative + false positive]) were calculated at multiple cut-off points. In addition, the area under the curve (AUC; the area under the receiver operating characteristic (ROC) curve) is considered to be a metric of fit between the true positive rate (sensitivity) and the false positive rate (1—specificity). AUC values can range from 0.5 to 1.0, with estimates closer to 1 indicating greater accuracy. Hanley and McNeil [36] suggested that a screener is a failure when an AUC is between 0.5 and 0.6, *poor* between 0.6 and 0.7, *fair* between 0.7 and 0.8, *acceptable* between 0.8 and 0.9, and *perfect* between 0.9 and 1.0.

## Results

### Discriminative validity

When applying the suggested cut-off score of ≥ 15 for a classification of ASD versus non-spectrum to all the samples, the AUC was 0.605, with a sensitivity of 0.677 and specificity of 0.593. When adjusting the cut-off score of the SCQ (e.g., cut-off ≥ 11 to ≥ 20), the AUC continued to reveal poor accuracy (range 0.581–0.634) with a better sensitivity when using a lower cut-off score (e.g., cut-off ≥ 11, sensitivity = 0.857, specificity = 0.315; Table 1) and a greater specificity when using a higher cut-off score (e.g., cut-off ≥ 20, sensitivity = 0.406, specificity = 0.759; Table 1).

**Table 1:** Prediction of ASD status based on Social Communication Questionnaire (SCQ)

**SCQ cut-off**	**AUC**	**Classification**^**a**^	**Sensitivity**	**Specificity**	**PPV**	**NPV**
**All (*N* = 187)**						
≥ 11	0.586		0.857	0.315	0.755	0.472
≥ 15	0.605	Poor	0.677	0.593	0.804	0.427
≥ 20	0.583		0.406	0.759	0.806	0.312
**Gender**						
**Males (*N* = 128)**						
≥ 11	0.539		0.838	0.241	0.753	0.350
≥ 15	0.631	Poor	0.675	0.586	0.818	0.395
≥ 20	0.597		0.400	0.793	0.842	0.324
**Females (*N* = 59)**						
≥ 11	0.612	Poor	0.867	0.357	0.591	0.714
≥ 15	0.686	Poor	0.800	0.571	0.667	0.727
≥ 20	0.624	Poor	0.533	0.714	0.667	0.588
**Age**						
**Age 4–7 years (*N* = 84)**						
≥ 11	0.627	Poor	0.873	0.381	0.809	0.500
≥ 15	0.587		0.698	0.476	0.800	0.345
≥ 20	0.508		0.444	0.571	0.757	0.255
**Age 8–11 years (*N* = 58)**						
≥ 11	0.553		0.833	0.272	0.652	0.500
≥ 15	0.638	Poor	0.639	0.636	0.742	0.519
≥ 20	0.649	Poor	0.389	0.909	0.875	0.476
**Age 12 years and above (*N* = 45)**						
≥ 11	0.563		0.853	0.273	0.784	0.375
≥ 15	0.702	Fair	0.676	0.727	0.885	0.421
≥ 20	0.586		0.353	0.818	0.857	0.290

*AUC* the area under the receiver operating characteristic (ROC) curve, *Sensitivity* number of true positive/(true positive + false negative), *Specificity* number of true negative/(true negative + false positive), *PPV* positive predictive value (number of true positive/[true positive + false positive]), *NPV* negative predictive value (number of true negative/[true negative + false negative])

^a^Hanley and McNeil [36] suggested that a screener is a failure when an AUC is between 0.5 and 0.6, *poor* between 0.6 and 0.7, *fair* between 0.7 and 0.8, *acceptable* between 0.8 and 0.9, and *perfect* between 0.9 and 1.0

When examining by sex, using the suggested cut-off score of ≥ 15, the SCQ achieved acceptable sensitivity among females with poor accuracy and specificity (*N* = 51; AUC = 0.686, sensitivity = 0.800, specificity = 0.571; Table 1), whereas AUC, sensitivity, and specificity remained poor in males (*N* = 136; AUC = 0.631, sensitivity = 0.675, specificity = 0.586). When adjusting the cut-off score of the SCQ (e.g., cut-off ≥ 11 to ≥ 20), the AUC among males (range 0.539–0.631) and females (AUC range 0.579–0.691) continued to reveal poor accuracy (Table 1).

To examine the discriminative validity of the SCQ with respect to age, the sample was separated into three age groups: age 4 to 7 years (*N* = 84), age 8 to 11 (*N* = 58), and age 12 and above (age range from 12.07 to 18.21; *N* = 45) based on previous literature (Corsello et al. 2007). In the youngest group, the AUC, sensitivity, and specificity were low when using the suggested cut-off score (cut-off ≥ 15; AUC = 0.587, sensitivity = 0.698, specificity = 0.476; Table 1), but the sensitivity improved when adjusting the cut-off to ≥ 11 (cut-off ≥ 11; AUC = 0.627, sensitivity = 0.873, specificity = 0.381; Table 1). For the older age groups, the 8 to 11 group had similar accuracy, sensitivity, and specificity to the whole group (cut-off ≥ 15; AUC = 0.638, sensitivity = 0.639, specificity = 0.636; Table 1), and age 12 and above yielded a fair AUC with better specificity (cut-off ≥ 15; AUC = 0.702, sensitivity = 0.676, specificity = 0.727; Table 1). All of the results were lower than reported in the original studies [1, 2].

### Comparison between groups

#### False negatives (FN) vs. true negatives (TN)

**Fig. 1:**
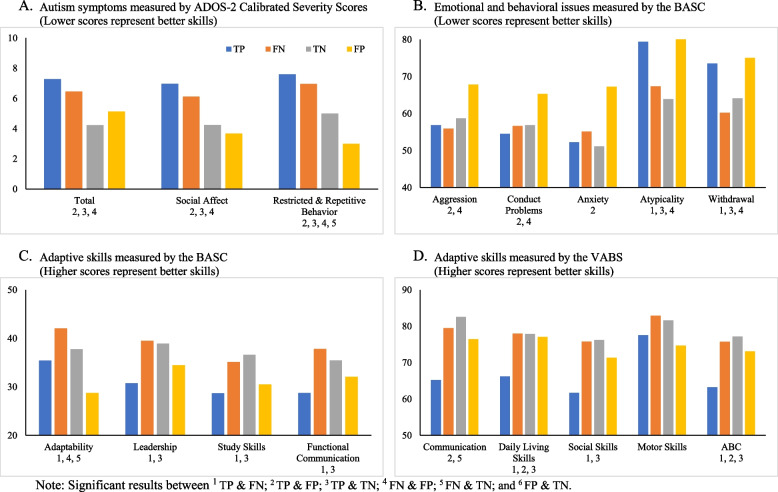
Group comparisons on autism symptoms severity, emotional and behavioral functioning, and adaptive skills

Compared to the FN, TN had more individuals who received ADHD, anxiety disorders, and depressive disorders as their final diagnoses, and more individuals with two or more comorbid diagnoses (Table 2). Regarding autism symptomatology, FN exhibited more restricted and repetitive behaviors than TN based on clinician observation (ADOS RRB CSS; Table 3, Fig. 1A), but caregivers’ reports (BASC Withdrawal and Atypicality) did not reveal significant differences among the two groups (Table 3, Fig. 1B). The adaptive skills assessed by the caregiver-reported BASC and VABS revealed similar results among FN and TN, suggesting similar adaptive skills among those misclassified and non-spectrum individuals (Table 3, Fig. 1C and D). Moreover, FN reportedly demonstrated better adaptability (i.e., the ability to adapt readily to changes in the environment) than TN on the BASC (Table 3, Fig. 1C). No significant differences on cognitive functioning, internalizing problems, or externalizing issues between groups (Table 3).

**Table 2:** Demographics for accurately and inaccurately classified cases

**Mean ± SD**	**True positives (TP)**	**False negatives (FN)**	**False positives (FP)**	**True negatives (TN)**	**Comparison**	
					**Chi-Square/*F* value**	**Post hoc analysis (Bonferroni or Fisher’s exact test)**
**Gender, *N* (%)**	**(*N* = 90)**	**(*N* = 43)**	**(*N* = 22)**	**(*N* = 32)**		
Male	65 (72.22)	29 (67.44)	14 (63.63)	20 (62.50)	1.37	
Female	25 (27.78)	14 (32.56)	8 (36.37)	12 (37.50)		
**Age (Mean ± SD)**						
Child	9.49 ± 4.64	9.33 ± 3.85	8.91 ± 3.23	9.24 ± 2.91	0.13	
**Comorbidity, *N* (%)**						
ADHD	41 (45.56)	8 (18.60)	11 (50.00)	21 (65.63)	18.78^c^	TN and FP > FN
Behavioral Disorders	1 (1.11)	1 (2.33)	6 (27.27)	1 (3.13)	17.88^c^	FP > TP, FN, and TN
Anxiety disorders	25 (27.78)	9 (20.93)	9 (40.91)	18 (56.25)	12.45^b^	TN > TP and FN
Depressive disorders	5 (6.10)	1 (2.33)	1 (4.55)	6 (18.75)	8.78^a^	TN > TP, FN, and FP
Language disorders	41 (45.56)	14 (32.56)	4 (18.18)	6 (18.75)	11.00^a^	TP > FP and TN
Learning disorders	1 (1.11)	1 (2.33)	1 (4.55)	3 (9.38)	5.43	
Intellectual disability	24 (26.67)	6 (13.95)	1 (4.55)	1 (3.13)	12.96^b^	TP > FP and TN
Any comorbidity^e^	77 (85.56)	22 (51.16)	16 (72.73)	28 (87.50)	15.68^b^	TP, FP, TN > FN

*p* value ^a^: *p* < 0.05; ^b^: *p* < 0.01; ^c^: *p* < 0.001; ^d^: *p* < 0.0001

^e^The individual received two or more diagnoses as the final diagnoses of their clinical evaluations

**Table 3:** Comparisons for accurately and inaccurately classified cases

**Mean ± SD**	**True positives (TP)**	**False negatives (FN)**	**False positives (FP)**	**True negatives (TN)**	**Comparison**	
					**Chi-square/*F* value**	**Post hoc analysis (Bonferroni or Fisher’s exact test)**
**Cognitive functioning (Mean ± SD)**	**(*N* = 90)**	**(*N* = 43)**	**(*N* = 22)**	**(*N* = 32)**		
Full-scale IQ	88.57 ± 23.67	89.97 ± 27.40	92.94 ± 12.74	103.33 ± 16.53	2.83^a^	TN > TP
Verbal IQ	86.61 ± 27.47	94.29 ± 23.19	83.06 ± 14.59	105.85 ± 16.56	4.29^b^	TN > TP
Non-verbal IQ	86.68 ± 27.28	89.87 ± 25.85	92.56 ± 16.03	101.93 ± 16.78	2.62^a^	TN > TP
**Symptoms severity (Mean ± SD)**						
**ADOS**						
ADOS Comparison Score	7.38 ± 2.54	6.84 ± 2.47	3.12 ± 2.91	4.22 ± 2.76	14.24^d^	TP > TN and FP, FN > FP
Social Affect CSS^e^	6.79 ± 2.27	6.29 ± 1.27	3.67 ± 2.12	4.24 ± 2.46	9.64^d^	TP > TN and FP FN > FP
Restricted and Repetitive Behavior CSS^e^	7.98 ± 2.43	6.97 ± 2.01	3.00 ± 2.45	4.12 ± 1.73	12.28^d^	TP & FN > TN and FP
**BASC**						
Externalizing problem	61.04 ± 12.54	59.77 ± 12.95	72.00 ± 18.56	68.55 ± 12.43	5.44^b^	FP > TP and FN
Internalizing problems	54.03 ± 10.12	56.86 ± 13.71	63.50 ± 13.51	60.70 ± 15.04	3.87^a^	FP > TP
Behavioral Symptoms Index	71.51 ± 11.87	65.43 ± 11.93	75.50 ± 10.72	70.32 ± 13.55	3.40^a^	FP > FN
Adaptive skills^f^	29.21 ± 8.49	37.91 ± 7.99	30.80 ± 5.03	34.83 ± 7.33	10.68^d^	FN > FP and TP, TN > TP
Hyperactivity	67.19 ± 12.61	64.14 ± 11.81	71.40 ± 12.63	69.09 ± 13.68	1.59	
Aggression	57.63 ± 13.85	56.74 ± 13.23	71.61 ± 20.10	66.70 ± 13.55	6.93^c^	FP > TP and FN
Conduct problems	54.59 ± 13.06	54.54 ± 14.24	68.56 ± 17.89	61.79 ± 11.18	5.56^b^	FP > TP and FN
Anxiety	54.07 ± 13.27	55.71 ± 14.69	62.65 ± 16.07	55.87 ± 13.55	3.17^a^	FP > TP
Depression	58.33 ± 11.89	59.91 ± 13.78	66.20 ± 13.41	65.74 ± 14.40	3.06	
Somatization	49.89 ± 9.41	51.57 ± 12.32	54.60 ± 13.49	55.04 ± 16.50	1.52	
Atypicality	78.34 ± 17.84	64.86 ± 15.40	77.00 ± 13.97	64.91 ± 13.05	7.89^d^	TP > TN and FN, FP > FN
Withdrawal	73.38 ± 12.95	62.51 ± 10.75	69.75 ± 14.45	58.83 ± 14.83	10.08^d^	TP > TN and FN, FP > TN
Attention	65.97 ± 8.90	63.00 ± 8.34	66.00 ± 6.18	65.57 ± 7.93	1.10	
Adaptability^f^	36.67 ± 8.39	42.49 ± 10.26	32.70 ± 5.80	34.96 ± 7.08	7.16^c^	FN > TP, FP, and TN
Social skills^f^	32.58 ± 10.01	40.00 ± 7.26	34.55 ± 8.60	37.00 ± 5.82	6.08^c^	FN > TP
Leadership^f^	31.78 ± 6.95	38.77 ± 6.21	34.00 ± 7.22	37.11 ± 6.81	7.23^c^	TN and FN > TP
Activities of daily living^f^	30.66 ± 10.72	38.00 ± 9.24	32.35 ± 6.74	37.57 ± 13.19	5.20^b^	TN and FN > TP
Functional communication^f^	29.03 ± 10.85	38.06 ± 9.90	32.85 ± 6.43	37.04 ± 9.37	8.08^d^	TN and FN > TP
**VABS**						
Communication	65.84 ± 18.13	75.90 ± 19.08	75.94 ± 12.52	81.83 ± 10.44	7.93^d^	TN and FN > TP
Daily living skills	67.86 ± 14.39	76.50 ± 14.46	78.00 ± 8.62	80.90 ± 10.51	9.07^d^	TN, FP, and FN > TP
Social skills	61.54 ± 13.02	73.24 ± 11.73	68.59 ± 10.79	76.72 ± 8.66	15.84^d^	TN and FN > TP
Motor skills	77.76 ± 12.04	81.57 ± 15.68	76.71 ± 20.84	89.38 ± 18.28	1.06	
Adaptive behavior composite	64.22 ± 12.73	73.93 ± 12.46	82.53 ± 7.32	77.59 ± 7.95	12.72^d^	TN, FP, and FN > TP

*p* value ^a^: *p* < 0.05; ^b^: *p* < 0.01; ^c^: *p* < 0.001; ^d^: *p* < 0.0001

^e^The Calibrated Severity Score (CSS) is a standardized version of ADOS-2 raw total scores aimed to minimize the impact of factors such as age, language, and cognitive ability

^f^Scores are reversed for the Adaptive Scales; lower scores represent poorer skills/performances, and higher scores reflect better skills/performances

#### False negatives (FN) vs. true positives (TP)

FN demonstrated similar comorbidity, cognitive functioning, and autism symptomatology with TP based on direct neuropsychological measures and clinicians’ observation on the ADOS-2 (Tables 2 and 3, Fig. 1A). Caregivers also reported similar internalizing and externalizing problems on the BASC among FN and TP (Table 3). Compared to TP, FN displayed less atypical behaviors and social withdrawal based on caregivers’ observation on the BASC (Table 3, Fig. 1B). FN also exhibited better adaptive skills then TP on the BASC and VABS (Table 3, Fig. 1C, D).

#### False positives (FP) vs. true positives (TP)

Compared to TP, FP had more individuals who were diagnosed with behavioral disorders, but fewer individuals received diagnosis of intellectual disability (Table 2). Consistent with their final diagnoses, TP displayed higher ADOS CSS than FP on SA, RRB, and total comparison score of ADOS (Table 3, Fig. 1A). However, caregivers of FP reported greater externalizing and internalizing problems than TP (Table 3). Specifically, caregivers of FP reported clinically significant scores on domains of hyperactivity, aggression, and conduct problems, and their scores on aggression and conduct problems were significantly higher than TP (Table 3, Fig. 1B). Caregivers of FP also reported greater internalizing problems, especially anxiety, which were significantly higher than TP group (Table 3, Fig. 1B). In general, caregivers of FP reported similar adaptive skills with TP across domains on the BASC and the VABS (Table 3, Fig. 1C, D), though FP reportedly displayed better daily living skills than TP on the VABS, resulted in greater overall Adaptive Behavioral Composite than TP (Table 3, Fig. 1D).

#### False positives (FP) vs. false negatives (FN)

Compared to FN, FP had more individuals who received ADHD and behavioral disorders as their final diagnoses among this sample, and more individuals with two or more diagnoses (Table 2). Consistent with their final diagnoses by the licensed psychologists, FN displayed higher ADOS CSS than FP on SA, RRB, and total comparison score of ADOS (Table 3, Fig. 1A). When examining internalizing and externalizing symptoms on the BASC, caregivers of FP reported greater behavioral issues than FN, including aggression and conduct problems (Table 3, Fig. 1B). The FP group also exhibited more atypical behaviors than FN (Table 3, Fig. 1B). In terms of adaptive skills, caregivers of FN and FP generally reported similar adaptive skills on the BASC and VABS (Table 3, Fig. 1C, D), with an exception that FN reportedly demonstrated better adaptability than FP based on the BASC (Table 3, Fig. 1C).

## Discussion

The present study examined the behavioral characteristics of children who were misclassified by the SCQ in a clinic-referred sample. Among the 187 participants in the present study, only 65% of individuals were accurately classified, revealing low sensitivity (0.68) and specificity (0.59). Individuals with ASD who were accurately classified on the SCQ (true positives) had lower adaptive skills and higher ADOS-2 scores than other groups, including false negatives. True positives did not differ from false negatives (or other groups) in terms of internalizing or externalizing behaviors. Individuals who were falsely classified as ASD (false positives) displayed more externalizing (e.g., aggression and conduct problems) and internalizing psychopathology (e.g., anxiety) and were more likely to be diagnosed with behavioral disorders (e.g., oppositional defiant disorder, conduct disorder) compared to the other groups. Regarding adaptive skills, while the non-spectrum group (true negatives and false positives) in general demonstrated higher adaptive skills than the ASD group (true positives and false negatives), the false negative group had a similar level of adaptive skills (e.g., communication, daily living skills, social skills, leadership, and functional communication) to the non-spectrum group.

Our results make an important contribution to knowledge about the limitations of classification properties of the SCQ in discriminating autism from other conditions in a referral population who seek their first diagnosis. Individuals who were falsely classified as ASD exhibited more externalizing (e.g., aggression and conduct problems) and internalizing issues (e.g., anxiety) compared to other groups. Moreover, they had comparable social impairments and atypical behaviors compared to children in the true positive group. It is likely that there may be a subset of children with more severe behavioral problems who also show early and continued social impairment, secondary to difficulty controlling impulses and regulating emotions. As a result, the caregivers may have endorsed numerous items on the SCQ to reflect the social challenges and general difficulties their children faced. Alternatively, another possibility is that parents are identifying atypical presentations in early development but are unable to clearly differentiate the types of difficulties they are observing on a yes/no questionnaire, thus reporting several social impairments that may better reflect current behavioral challenges. Since these behavioral challenges are not specifically differentiated and measured on the SCQ, the reporters may not have adequate places to voice their concerns, resulting in elevated scores. It is important to note that anxiety and depression in young children can be difficult for parents to differentiate from autistic behaviors, as internalizing symptoms may present as social withdrawal, irritability, rigidity, preference for routine and order, and repetitive behaviors, which are also symptoms of ASD [37, 38]. In fact, there is a known challenge of differentiating autistic symptomology from developmental and behavioral presentations, even when utilizing a standardized diagnostic measure such as the ADOS by trained clinicians [39]. Using the SCQ in combination with other measures for internalizing and externalizing psychopathology is recommended [40–43].

Another notable finding was that the false negative group appeared to display fewer developmental and adaptive impairments compared to the true positive group, suggesting that the accuracy of the SCQ may be highly influenced by developmental factors. Children in the false negative group clearly exhibited ASD symptoms, and their ADOS-2 scores did not differ from those of the true positive group, but their presentations may have been subtler than those accurately captured with ASD by the SCQ or may be masked by their better-developed adaptive skills. Therefore, their caregivers were not able to reflect their observations on the SCQ. Our results corroborate previous findings that the SCQ may miss individuals with less overall impairment [5]. Moreover, as a parent-report questionnaire, the SCQ highly relies on the reporter’s knowledge, experiences, observation, and understanding of autistic characteristics. Previous studies [44, 45] suggested that if a parent already has a prior understanding of ASD or knows their child has ASD, they are more likely to differentiate core autism symptoms from other challenging behaviors on questionnaires or endorse ASD symptoms to a greater degree, revealing the reporters’ knowledge influences how they respond to the screening questions. Parental concerns about their children’s development have also been suggested to influence parent-report instrument performance [46]. It is possible that the SCQ could vary according to parental understanding of ASD and level of concern. Future research is needed to determine the optimal ways to screen for ASD in children where parents have minimal concerns about their children’s development.

Previous studies utilizing clinic-referred samples have suggested different cut-off scores according to age and purpose to increase accuracy [5, 10, 14]. Consistent with the literature, the current findings revealed suboptimal sensitivity and specificity of the SCQ, lower than those reported in the original study [2] as well as subsequent studies [5, 14]. However, adjusting cut-off scores based on age and sex did not improve the accuracy of the SCQ to a satisfactory level or impact the specificity of the measure on this sample. Our findings suggest that age and sex may not be the critical factors that impact the accuracy and usefulness of the SCQ among clinic-referred samples. Given our results suggesting suboptimal sensitivity and specificity of the SCQ, it is important to highlight for clinicians and researchers that the SCQ has limitations in accurately identifying children who will go on to receive an ASD diagnosis, particularly for children with complex presentations and other co-occurring mental health and behavioral diagnoses. Therefore, the SCQ used in isolation seems to have limited utility in assisting with the clinical triaging process or serving as a screening tool for inclusion or exclusion criteria in the research setting. This is problematic, given that there is a severe shortage of professionals who are trained to provide specialized evaluations for ASD, leaving specialty ASD clinics with long waiting lists, barriers to intervention access, and increased parental stress [47–49]. Tools that are validated in accurately classifying young children are critically necessary [50–52].

Several limitations of the present study should be addressed in future research. Although we had a relatively large sample, especially for a clinic-referred population that underwent gold-standard measures for ASD diagnosis, our sample sizes were made smaller when stratifying by age, sex, and SCQ status. In addition, selection bias could potentially limit the generalizability of our results. We were unable to explore additional family factors that may contribute to the sensitivity and specificity of the SCQ (e.g., primary language, race/ethnicity, and educational level) due to the limited inclusion of diverse families within our sample and inconsistent demographic information provided by families during the clinical intake process. It is also important to note that the SCQ score was available for the clinicians at the time of making a clinical diagnosis, and the clinical impression and diagnostic classification may not be independent. Further, the comorbidity of this clinic-referred sample was relatively high across the groups. It suggested that this is a complex sample, which might be contributing to the modest classification accuracy of the SCQ. The results may not generalize to other clinically referred samples with different clinical features. Future work should replicate our results in larger samples and explore additional factors that may contribute to individual differences related to the sensitivity and specificity of the SCQ.

In summary, the present study examined the sensitivity and specificity of the SCQ by assessing a clinical sample of children and adolescents with and without ASD. This study further examined the individual differences in sensitivity and specificity by describing the characteristics of children misclassified using the SCQ. These findings suggested that the SCQ may be less useful for young children with internalizing and externalizing symptoms and other more complex clinical presentations, highlighting the importance of taking an individual difference and multi-informant approach for ASD assessment. Clinically, these results underscore the necessity of comprehensive evaluations for children, particularly for internalizing and externalizing psychopathology in addition to autistic symptoms, to promote optimal diagnostic and intervention outcomes. In research settings, our results suggest that solely relying on the SCQ for inclusion criteria may be risky, especially for families who are less familiar with ASD. Future studies will be needed to determine if SCQ performs best in population-based screening (e.g., school-based screening) or clinically referred samples, as well as whether parental awareness of ASD impacts the reporting pattern on the SCQ, further influencing the sensitivity and specificity of the SCQ within certain populations.

## Conclusions

Our findings make an important contribution to the literature on ASD measurement by specifying the behavioral characteristics of children misclassified by the SCQ. Specifically, individuals who were falsely classified as ASD by the SCQ exhibited more externalizing (e.g., aggression and conduct problems) and internalizing issues (e.g., anxiety), and they were more likely to be diagnosed with behavioral disorders (e.g., oppositional defiant disorder and conduct disorder) compared to other groups. The false negative group appeared to display fewer developmental and adaptive impairments compared to the true positive group. These findings suggest that the accuracy of the SCQ is highly influenced by developmental and behavioral factors, and the SCQ may be less useful for young children with internalizing and externalizing symptoms and other more complex clinical presentations. Clinicians and researchers could consider using the SCQ in combination with other assessment tools for internalizing and externalizing psychopathology.

## Data Availability

The datasets and unpublished materials used and/or analyzed in this study are available from the corresponding author on reasonable request.

## Abbreviations

ASD: Autism spectrum disorder
IQ: Intelligence quotient
ADHD: Attention-deficit hyperactivity disorder
TP: True positives
FN: False negatives
FP: False positives
TN: True negatives
ADOS-2: Autism Diagnostic Observation Schedule, Second Edition
ADI-R: Autism Diagnostic Interview, Revised
FSIQ: Full Scale Intelligence Quotient
BASC-PRS: Behavioral Assessment System for Children, Parent Rating Scale
VABS: Vineland Adaptive Behavior Scale
AUC: Area under the receiver operating characteristic curve
ROC: Receiver operating characteristic
RRB: Restricted and Repetitive Behavior
CSS: Calibrated Severity Scores
